# Advancements in innate immune regulation strategies in islet transplantation

**DOI:** 10.3389/fimmu.2023.1341314

**Published:** 2024-01-15

**Authors:** Kehang Duan, Jiao Liu, Jian Zhang, Tongjia Chu, Huan Liu, Fengxiang Lou, Ziyu Liu, Bing Gao, Shixiong Wei, Feng Wei

**Affiliations:** ^1^ Department of Hepatobiliary and Pancreatic Surgery, General Surgery Center, The First Hospital of Jilin University, Changchun, Jilin, China; ^2^ Department of Cardiology, The Second Hospital of Jilin University, Changchun, Jilin, China

**Keywords:** islet transplantation, innate immune response, immunoregulation, macrophage, diabetes

## Abstract

As a newly emerging organ transplantation technique, islet transplantation has shown the advantages of minimal trauma and high safety since it was first carried out. The proposal of the Edmonton protocol, which has been widely applied, was a breakthrough in this method. However, direct contact between islets and portal vein blood will cause a robust innate immune response leading to massive apoptosis of the graft, and macrophages play an essential role in the innate immune response. Therefore, therapeutic strategies targeting macrophages in the innate immune response have become a popular research topic in recent years. This paper will summarize and analyze recent research on strategies for regulating innate immunity, primarily focusing on macrophages, in the field of islet transplantation, including drug therapy, optimization of islet preparation process, islet engineering and Mesenchymal stem cells cotransplantation. We also expounded the heterogeneity, plasticity and activation mechanism of macrophages in islet transplantation, providing a theoretical basis for further research.

## 1 Introduction

Since the Edmonton protocol was proposed in 2000, islet transplantation has developed rapidly as a new technology. This protocol proposed a glucocorticoid-free immunosuppressive regimen consisting of sirolimus, tacrolimus, and daclizumab and a scheme of sequential transplantation of islets from at least two donor pancreases. During the follow-up period (with a median follow-up time of 1 year), all seven patients who received this protocol achieved insulin independence (1). Subsequent clinical trials verified the protocol’s effectiveness and noted the necessity of minimizing the interval between 2 islet infusions (2). The outcomes of islet transplantation over the past two decades were assessed in a large cohort study conducted by the Edmonton group. Among 255 patients, the 5-year insulin independence rate after operation was 32%, and 8% still had insulin independence 20 years later (3). Islet transplantation has the advantages of minimal trauma, high safety, a short hospital stay, less patient suffering, and repeatability. It is especially suitable for “brittle diabetes” type I diabetes patients, type II diabetes patients with pancreatic islet dysfunction, diabetes patients after liver and kidney transplantation, and patients with nonmalignant pancreatic resection for the prevention of postoperative diabetes. Compared with traditional drug and insulin therapy, animal experiments have proven that islet transplantation can increase the body and muscle weight of diabetic rats, more effectively reduce proteinuria, significantly improve the conduction velocity of the tail nerve, restore thermal and ameliorate mechanical nociceptive thresholds, and improve the residual β-cell state in the recipient pancreas (4). In a clinical control experiment, islet transplantation slowed the progression of diabetic microvascular complications, such as a declining renal glomerular filtration rate and retinal changes (5); compared with no transplantation, islet transplantation resulted in near-normal platelet activation and prothrombotic factor levels, cerebral metabolism and function, and neuropsychological test results (6).

The most common and ideal transplantation route is through the transhepatic portal vein (7). Nevertheless, islet cells implanted in the portal vein are often lost within three days due to hypoxia, nutritional deficiency, the mechanical pressure of the portal vein, and the immediate blood-mediated inflammatory response (IBMIR). The early loss of implanted islets is an essential factor in the failure to achieve insulin independence in single transplant patients. The IBMIR was first proposed by the W Bennet team in 1999 and was validated in *in vivo* and *in vitro* experiments. The reaction is defined as a rapid, strong, and nonspecific immune inflammatory response induced by tissue factors exposed on the surfaces of islets, with characteristics such as platelet activation, aggregation, coagulation, and complement system activation, as well as an infiltration of neutrophils, monocytes and macrophages and release of inflammatory factors. Usually, within 15 minutes after transplantation, the islets are encircled by a thrombus; after 1 hour, the islets undergo massive apoptosis due to the infiltration of white blood cells (8). This reaction also leads to early apoptosis of autologous islets in patients who undergo total pancreatectomy and autologous islet transplantation (9). At present, the standard clinical therapy relies on anticoagulants such as heparin, soluble complement receptor 1, and protease inhibitors such as ulinastatin. However, the IBMIR is a complex reaction involving multiple factors, and 50-70% of transplanted islets still undergo early apoptosis due to the IBMIR in existing therapeutic schemes. Therefore, the inhibition of the IBMIR to avoid early apoptosis of transplanted islets is an urgent issue in clinical work (10).

Because macrophages are essential in the IBMIR and subsequent adaptive immunity and are plastic and heterogeneous in inflammatory reactions (11, 12), This article reviews the strategy of innate immune regulation with macrophages as the primary target in recent years in islet transplantation to provide references for subsequent basic research and improve the survival rate of islets in clinical islet transplantation (**Tables 1**, **2**).

**Table 1:** *In vivo* and *in vitro* experiments for all therapeutic strategy.

Therapeutic strategy	vitro studies	vivo studies in mammals	Donor	Receptor	Transplantation type
PTD (13)	+	+	mice	mice	Allotransplantation
CO (14)	+	+	mice	mice	Allotransplantation
NOX-A12 (15)	–	+	mice	mice	Isotransplantation
mNOX-E36 (15)	–	+	mice	mice	Isotransplantation
MCC950 (16)	+	+	mice	mice	Isotransplantation
hAAT (17) (18)	+	+	mice	mice	Allotransplantation
			mice	mice	Allotransplantation
			human	mice	Xenotransplantation
1,25(OH)2D3 (19–21)	+	+	rat	rat	Isotransplantation
DHMEQ (22)	+	+	mice	mice	Isotransplantation
ARA290 (23)	+	+	mice	mice	Isotransplantation
liraglutide (24) (25)	+	+	rat	rat	Isotransplantation
teduglutide (26)	+	–	N/A	N/A	N/A
captopril (27)	+	+	pig	mice	Xenotransplantation
Diannexin (28)	+	+	mice	mice	Isotransplantation
CP-ASCs (29)	+	+	mice	mice	Isotransplantation
			human	mice	Xenotransplantation
autologous MSCs (30)	+	+	human	human	Allotransplantation
MSCs-derived exosomes (31)	+	+	rat	mice	Xenotransplantation
OptiPrep (32)	+	+	human	mice	Xenotransplantation
APT070 (33)	+	+	human	mice	Xenotransplantation
anakinra (34)	+	–	N/A	N/A	N/A
ATF3 KO (35)	+	+	mice	mice	Isotransplantation
overexpress Del-1 (36)	+	+	mice	mice	Isotransplantation
Overexpress sTNF-αR-Fc/HO-1 (37)	+	+	pig	mice	Xenotransplantation
MHC I and II KO and overexpress CD47 (38)	+	+	human	mice	Xenotransplantation
			mice	mice	Allotransplantation
			mice	mice	Isotransplantation
thermoplastic polyurethane-based nanofiber capsules (39)	+	+	mice	mice	Isotransplantation
	+	+	mice	mice	Allotransplantation
Dexa (40)	+	+	pig	mice	Xenotransplantation
bilirubin (41)	+	+	mice	mice	Isotransplantation
IDO (42)	+	+	rat	mice	Xenotransplantation
TA (43–45)	+	+	mice	mice	Allotransplantation

PTD, protein transduction domain proteins; 1,25(OH)2D3, 1,25-Dihydroxy vitamin D3; DHMEQ, Dehydroxymethylepoxyquinomicin.

**Table 2:** Mechanisms and limitations of all therapeutic strategy.

Therapeutic strategy	mechanism	limitations
PTD (13)	Block TLR4 signaling	Risk of affecting islet vascularization; neglect the impact of clinical immunosuppression schemes
CO (14)	Block TLR4 signaling	
NOX-A12 (15)	Bind and antagonize CCL2/MCP-1	neglect the impact of clinical immunosuppression schemes
mNOX-E36 (15)	Bind and antagonize CXCL12/SDF-1	
MCC950 (16)	Inhibit the activation of NLRP3 inflammasome	Risk of affecting islet vascularization; neglect the impact of clinical immunosuppression schemes
hAAT (17) (18)	Increase IL-1Ra expression and secretion Inhibit IFN-γ-induced STAT1 phosphorylation; Inhibit iNOS production	neglect the impact of clinical immunosuppression schemes
1,25(OH)2D3 (19–21)	Reduce TNF-α/NF-κB pathway activation; Reduce macrophage recruitment; Promote the polarization of M1 macrophages into M2 macrophages via the VDR-PPARγ pathway	neglect the impact of clinical immunosuppression schemes; Risk of graft fibrosis
DHMEQ (22)	Inhibit NF-κB activation at the nuclear translocation level of macrophage-based immune cells	neglect the impact of clinical immunosuppression schemes
ARA290 (23)	Inhibit NF-κB pathway by activating EPOR-βcR/PI3K-Akt signaling pathway	neglect the impact of clinical immunosuppression schemes
liraglutide (24) (25)	Inhibit the expression of proinflammatory cytokines; Inhibit macrophage recruitment; modulate macrophages M2 polarization via the cAMP-PKA-STAT3 signaling pathway	Risk of affecting islet vascularization; neglect the impact of clinical immunosuppression schemes
teduglutide (26)	Inhibit M1 macrophages polarization by mediating the crosstalk between endocrine cells and macrophages	
captopril (27)	Protect ECM by inhibiting gelatinase activity to reduce macrophage infiltration	Risk of affecting islet vascularization
Diannexin (28)	Inhibit leukocyte and platelet aggregation attachment by binding externalized phosphatidylserine residues on the surface of early apoptotic cells	Risk of affecting islet vascularization
CP-ASCs (29)	Mediate the expression of TNF receptor superfamily member 11b through paracrine IGF-1; Reduce the infiltration of macrophages into the graft	Risk of induced thrombosis in the liver; difficult to ensure the close contact between MSCs and islets
MSCs-derived exosomes (31)	Regulate macrophages by regulating NF-κB signaling pathway	The total amount of drugs carried by microcapsules is limited
OptiPrep (32)	Reduce the production of cytokines/chemokines during islet preparation.	Compared with Ficoll-based purification, Islet yields decreased slightly (have no statistical differences)
APT070 (33)	Reduce iC3b production in islets; Reduce C4d and C5b-9 deposition in islets	Unable to target graft administration posttransplantation
anakinra (34)	Reduce the formation of hIAPP	Risk of subcutaneous amyloidosis caused by long-term subcutaneous injection
ATF3 KO (35)	Inhibit the expression of proinflammatory cytokines and chemokines in islets	Potential cytotoxicity and tumorigenicity
overexpress Del-1 (36)	Inhibit platelet-monocyte aggregate formation by blocking the interaction between monocyte Mac-1-integrin and platelet GPIb	
overexpress sTNF-αR-Fc/HO-1 (37)	Inhibit the expression of proinflammatory cytokines and chemokines in islets; Inhibit macrophage recruitment	
MHC I and II KO and overexpress CD47 (38)	Inhibit innate and adaptive immunity	
thermoplastic polyurethane-based nanofiber capsules (39)	Block macrophages activation	Potential cytotoxicity and tumorigenicity
Dexa (40)	Reduce macrophage-dominant inflammatory cell infiltration and pericapsular fibrosis	The total amount of drugs carried by microcapsules is limited
bilirubin (41)	Activate the Nrf2 pathway to polarize macrophages to the M2 phenotype; Inhibit the NF-κB pathway to inhibit M1 polarization	
IDO (42)	Induce tryptophan deficiency; Reduce the proinflammatory activity and viability of macrophages; Reduce the infiltration of macrophages in the graft	
TA (43–45)	Regulate macrophages polarization by reducing the production of ROS	

PTD, protein transduction domain proteins; 1,25(OH)2D3, 1,25-Dihydroxy vitamin D3; DHMEQ, Dehydroxymethylepoxyquinomicin.

## 2 Activation and role of macrophages in the IBMIR

Clinical islet transplantation requires four steps: perfusion of the donor pancreas, digestion of the pancreas to separate the islets from exocrine glands, purification of the islets, and transplantation into recipients through the portal vein (7). When the prepared islets are infused into patients via the portal vein, it will trigger IBMIR.

IBMIR is initiated by a strong activation of the coagulation cascade. After contact with blood in the portal vein, islet tissue factor expression induces the extrinsic coagulation pathway. The negative charge on the islet surface triggers the intrinsic coagulation pathway. At the same time, islets secrete inflammatory factors such as IL-8 and MCP-1, which have chemotactic and proinflammatory effects on macrophages and neutrophils (46, 47).

Activated platelets may attach through binding to extracellular matrix (ECM) and collagen on the surface of the islet. Meanwhile, owing to the fast and transient expression of p-selectin on the membrane of activated platelet alpha granules and vascular endothelial Weibel-Palade bodies, the p-selectin lectin-like domain binds to sialyl Lewis x and the p-selectin glycoprotein ligand 1 in the neutrophils and mononuclear cells, thus mediating the rolling of neutrophils and mononuclear cells on the endothelial cell surface and the adhesion of neutrophils and mononuclear cells to platelets (48, 49). On the other hand, vascular endothelial cells secrete IL-6 and IL-8 to promote the aggregation of neutrophils and macrophages (46).

Complement activation is triggered by the natural immunoglobulins IgG and IgM. When isolated islets are exposed to blood, the complement system is quickly activated, leading to the lysis of islet cells. At the same time, the production of the allergic toxins C3a and C5a further induces the aggregation of macrophages and neutrophils and promotes mononuclear cells to release cytokines such as IL-1, IL-6, IL-8, and TNF-α (50).

**Figure 1:**
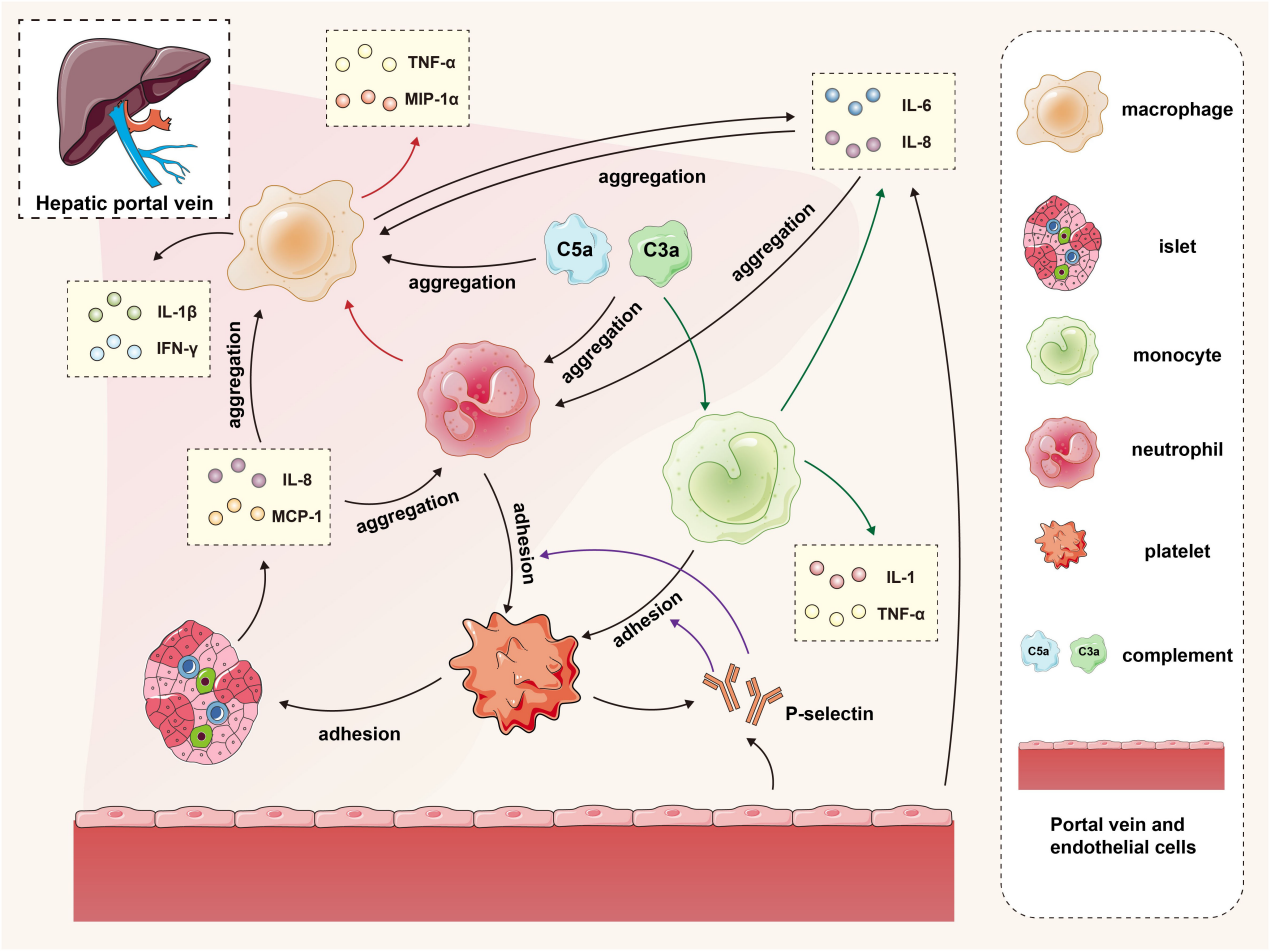
The interaction of islets, macrophages, monocytes, neutrophils, platelets, complement and endothelial cells in the IBMIR. In the early stage of transplantation, islets secrete proinflammatory factors and activate the complement system, promoting the recruitment of platelets, neutrophils, and mononuclear macrophages to the graft. Vascular endothelial cells secrete cytokines while releasing P-selectin, facilitating adhesion between monocytes/neutrophils and platelets. The accumulated mononuclear macrophages and neutrophils further contribute to macrophage recruitment and cytokine secretion. The green arrows represent the process by which complement promotes monocytes to secrete cytokines, the red arrows depict the process by which neutrophils promote macrophages to secrete cytokines, and the purple arrows illustrate the procedure in which P-selectin facilitates adherence of monocytes and neutrophils to platelets.

Granulocytes appear 8 hours after islet transplantation and extensively infiltrate the transplants after 12 hours. Neutrophils are predominant members of the granulocyte family and the first line of defense of innate immunity. They contain many cytokines, which are released when activated and have destructive effects on islets; neutrophils make significant contributions to the activation and recruitment of macrophages in acute inflammation sites. After activation, they produce various chemokines to attract mononuclear cells and macrophages; in addition, the infiltration of neutrophils leads to the release of cytokines from T cells and macrophages, such as tumor necrosis factor α (TNF-α) and macrophage inflammatory protein 1α. The mobilization of this immune effector could not only expand IBMIR but also induce subsequent adaptive immunity, inducing and enhancing cellular rejection responses (50, 51) (**Figure 1**).

The gathered macrophages continue to secrete cytokines such as IL-6 and IL-8 to maintain the inflammatory response and release proinflammatory factors such as IL-1β, IFN-γ, and TNF-α. The IL-1β secreted by macrophages and neutrophils binds to the IL-1β receptor on the surface of islet cells, activating the IL-1 receptor-associated kinase to activate TNF receptor-associated factor 6, which leads to the phosphorylation and degradation of IκB, releasing NF-κB from the inhibitory IκB, and entering the nucleus of cells to regulate the transcription of various genes, including IL-1, IL-6, TNF-α and iNOS. The TNF-α produced by macrophages and islet cells binds to the TNF receptor, activating the NF-kB and MAPK pathways and inducing cell apoptosis. Apoptosis is activated by the activation of caspase-3, mediated by the MAPK pathway, or by activating effector caspases, including caspase-3, which FADD mediates. The interferon-γ (IFN-γ) produced by macrophages binds to IFN-γ receptors on the surface of the islets, activating JAK1 and JAK2. Activated JAK2 activates signal transducers and activators of transcription 1 (STAT1). Then, STAT1 is transferred to the nucleus for gene regulation, eventually leading to islet apoptosis. The apoptosis-promoting effect of STAT1 may be partially mediated by the activation of caspase-2, caspase-3 and caspase-7 (52). Under the combined effects of the cytokines IL-1β, TNF-α, and IFN-γ, overexpression of iNOS in β cells and macrophages leads to excessive NO synthesis. Subsequently, NO loses an electron to combine with superoxide free radicals, forming a highly active free radical peroxynitrite (ONOO-). The cytotoxicity of ONOO- then induces apoptosis in islet cells. On the other hand, macrophages play an antigen-presenting role, promoting the activation of T cells into CD8+ T cells and CD4+ T cells. Activated T cells produce cytokines such as IFN-γ, TNF-α, and lymphotoxin, thereby inducing β-cell apoptosis. T cells also express Fas receptor-associated ligands and TNF-related apoptosis-inducing ligands, activating effector caspases to cause cellular apoptosis. In addition, CD8+ T cells directly contact and promote the release of granzyme B into the cytoplasm of target cells through perforin, thereby activating nucleases and caspases to kill target cells (11, 50, 53).

## 3 Macrophage origins in islet transplantation

Macrophages play crucial roles in the IBMIR after islet transplantation. Generally, they aggregate around the islets at 8 hours and infiltrate them at 12 hours posttransplantation. By 24 hours posttransplantation, the transplanted islets are entirely infiltrated (51). In islet transplantation, macrophages usually come from three sources: Kupffer cells (KCs) in the recipient’s liver, mononuclear macrophages from the recipient’s bone marrow, and macrophages resident in the donor’s islets.

For donor-derived macrophages, on the one hand, islet-resident macrophages mediate the production of islet interleukin (IL)-1β and impair the function of beta cells induced by islet amyloid-like polypeptides (54). On the other hand, some studies have shown that during islet compensation in the early stage of diabetes, islet-resident macrophages contribute to angiogenesis by supporting islet vascular endothelial growth factor A (VEGF-A) secretion during islet remodeling, suggesting their critical role in supporting islet compensation during diabetes (55). Further animal experiments confirmed that in a mouse autoimmune diabetes model, islet resident macrophages showed different phenotypes, such as maturation, self-replication, proinflammation, and immune tolerance, throughout the disease course, demonstrating heterogeneity in the inflammatory process (56). Although the number of donor-derived macrophages is usually small, their diverse functions in islet cells during the diabetes stage prove their importance. However, relevant research on donor-derived macrophages in islet transplantation is still lacking.

KCs are resident macrophages in the liver. KCs in the sinusoids can phagocytose pathogens from the arterial and venous systems, playing an essential role in innate immunity. Single-cell RNA sequencing analysis of freshly isolated human liver demonstrates the presence of two distinct intrahepatic CD68+ macrophage subsets in the steady state: one is an inflammatory macrophage subset enriched in the expression of LYZ, CSTA, and CD74; the other is a tolerogenic macrophage subset distinguished by high expression of CD5L, MARCO, and VSIG4. Two distinct cell populations under the same lipopolysaccharide (LPS)/IFN-γ stimulation conditions showed that the tolerogenic function subset secreted less TNF-α (57). In another study, F4/80+ KCs in the mouse liver were divided into CD68+ subsets with phagocytic activity and CD11b+ subsets with cytokine generation capability (58). KCs preferentially induce tolerogenic immunity under noninflammatory conditions and perceive the condition of liver tissue, and their response to environmental changes plays an essential role in the pathogenesis of liver diseases (59). In the development of alcohol-related liver disease, KCs induce oxidative stress and inflammation in the liver and promote the progression of alcohol-related liver disease by participating in reactive oxygen species (ROS) production and activating pathways leading to cytokine and chemokine production. The liver stellate cells activated by KCs contribute to the progression of liver fibrosis. In islet transplantation, implanted islet cells, acinar cells, and secreted soluble factors activate KCs to secrete proinflammatory cytokines, such as IL-1β and TNF-α. The inhibition of KCs may extend the survival of implanted islets (60, 61). In summary, KCs exhibit significant heterogeneity and strong plasticity under physiological and inflammatory conditions.

Monocytes are generally thought to be derived from myeloid progenitors derived from pluripotent hematopoietic stem cells in the bone marrow. Monocytes further differentiate into dendritic cells, macrophages, and osteoclasts. In humans, monocytes can be divided into the CD14hiCD16- classic subtype, which is mainly responsible for innate perception, immune response, migration, and differentiation into macrophages at the injury site, and CD14loCD16+ nonclassic monocytes, which are mainly responsible for vessel system monitoring and tissue repair. The two subtypes are analogous to the CX3CR1intCCR2+CD62L+CD43loLy6Chi inflammatory subtype and CX3CR1hiCCR2-CD62L-CD43hiLy6Clo patrolling monocytes found in mouse tissues. In addition, a small number of CD14+CD16+ “intermediate” subtype monocytes are mainly responsible for antigen presentation and cytokine secretion during the immune response and play an essential role in the inflammatory cascade, also known as transitional inflammatory monocytes (62–64). Under inflammatory conditions, such as islet transplantation within the portal vein, islets and endothelial cells release inflammatory factors such as CCR2, recruiting “classic” monocytes out of the bone marrow to the inflammatory sites and differentiating into dendritic cells and inflammatory macrophages, producing TNF, iNOS and ROS to trigger and expand the inflammatory response; “nonclassic” monocytes usually differentiate into M2 immunomodulatory phenotype macrophages while suppressing inflammation, thus promoting the vascularization of the transplanted islets and allowing the graft to colonize the hepatic sinuses and survive for a long time to perform islet functions (41). As essential participants in the innate immune response, monocyte-derived macrophages are also highly plastic and show “cross-differentiation” under the influence of different environments, which has been confirmed in the inflammatory environment of different diseases (62). In islet transplantation, depleting dendritic cells derived from recipient monocytes can enhance the early graft function (65). Another study demonstrated that bone marrow-derived mononuclear cells can be cultured into spheroids, with CXCR4+CD31+ myeloid cells being the main cell components. In the islet transplantation model under the renal capsule of syngeneic mice, cotransplantation of bone marrow–derived spheroids improves the blood supply reconstruction and graft function (66). Although research on macrophages from different sources in innate immune response in the field of islet transplantation remains limited, existing studies have provided initial insights into the influence of monocytes with different functions on islet transplantation.

## 4 Advances in macrophage studies in islet transplantation

Due to the importance of macrophages in the immune response to islet transplants, research on macrophages has always been a popular topic in the field of islet transplantation, and considerable progress and achievements have been made. Most of them focus on traditional drug therapy, interstitial cell coculture or cotransplantation, the optimization of islet isolation and culture methods, islet modification and engineering before transplantation, etc. The most common and effective strategy is to directly regulate the innate immune response of the recipient using traditional drugs to reduce damage to the graft caused by immune cells, including macrophages.

### 4.1 Drug therapies targeting macrophages in the innate immune response

Toll-like receptors (TLRs) are important sensors for innate immunity and bridges between innate and adaptive immunity (67). Mammalian TLRs that occupy the plasma membrane include those that detect microbial cell surface components, such as TLR4 (LPS), TLR5 (flagellin), and TLRs 1, 2 and 6 (bacterial lipoproteins). TLRs found in endosomes detect nucleic acids, such as TLR3 (double stranded (ds) RNA), TLR7 and 8 (single stranded (ss) RNA), and TLR9 (unmethylated CpG containing ssDNA) (68). TLR4 is a highly representative TLR, and its expression in islets is controversial. Studies have shown that TLR4 is not expressed in mouse islet β cells and that islet resident macrophages are its major source and mediate the TLR4 pathway to induce proinflammatory factor secretion in the islets (69). Other related studies suggest that TLR4 and its related molecules, myeloid differentiation protein-2 and the endotoxin receptor CD14, are expressed in islet β cells (70, 71). Regardless of the source of TLR4, the islet isolation and transplantation process can lead to the upregulation of TLR4 expression in the islets, the activation of the TLR4/MyD88 pathway, and the production of chemokines that recruit mononuclear cells and macrophages to the islets. By blocking TLR4 activation with carbon monoxide, protein transduction domain proteins, etc., inflammation and macrophage infiltration during transplantation can be suppressed (13, 14). Inhibiting the functions of the chemokines produced by islet cells can effectively reduce macrophage infiltration of the graft.

L-selectin, also known as Spiegelmers, is a new class of oligonucleotide drug. Two specific L-selectins, mNOX-E36 and NOX-A12, bind and antagonize CCL2/MCP-1 and CXCL12/SDF-1, respectively. In a syngeneic intraportal transplant mouse model, mNOX-E36 and NOX-A12 decreased the hepatic recruitment of inflammatory monocytes, CD11b+/Ly6Chi/CCR2+ cells and CD11b+/Ly6Chi/CXCR4+ cells; prevented inflammation-mediated islet destruction; and significantly improved islet function after transplantation (15).

In the islet isolation process, detrimental factors such as damage-associated molecular patterns, ROS, oxidative stress, and mitochondrial dysfunction can activate the NLRP3 inflammasome within islet cells, and the activated inflammasome cleaves caspase-1 and activates pro-IL-1β to IL-1β. IL-1β can induce macrophage recruitment to the transplant, upregulate the Fas receptor, activate the NF-κB pathway, and cause β-cell apoptosis and functional impairment. MCC950 is a specific inhibitor of the NLRP3 inflammasome that can inhibit IL-1β expression in transplant islets, the infiltration of macrophages around the islets, and fluctuations in blood glucose in the recipient (16).

Another strategy to target the proinflammatory effects of IL-1β is to block the activation of its corresponding receptor. Interleukin-1 receptor antagonist (IL-1Ra) is an endogenous IL-1 inhibitor that can bind to IL-1R1, prevent IL-1R accessory protein recruitment, and inhibit the activation of IL-1R. Human alpha1-antitrypsin (hAAT) is a serine protease inhibitor with tissue protection, anti-inflammatory, and immunoregulation activities. hAAT increased IL-1Ra expression and secretion both in primary islet and macrophages. In a mouse model of renal subcapsular islet allotransplantation, hAAT pretreatment significantly increased insulin transcription levels, while the transcription levels of IL-1β, TNF-α, and other inflammatory factors in islet grafts significantly decreased (17). Moreover, hAAT partially inhibits M1-type macrophage activation by inhibiting IFN-γ-induced STAT1 phosphorylation and iNOS production (18).

1,25-Dihydroxy vitamin D3, also known as calcitriol, is commonly converted from vitamin D3 in the human body. Its effects on the innate and adaptive immune systems are manifested as the induction of immunological tolerance and the activation of anti-inflammatory pathways. Its main functions are as follows: 1. to inhibit the synthesis of proinflammatory cytokines by monocytes and macrophages; 2. To reduce the expression of major histocompatibility complex-II class molecules on the surface of macrophages, thereby reducing the antigen presentation and T-cell stimulation ability of macrophages; and 3. to promote the polarization of macrophages from the M1 phenotype to the M2 phenotype via the VDR-PPAR*γ* pathway. Calcitriol may prolong the survival of homologous islet grafts by reducing TNF-α/NF-kB pathway activation and macrophage recruitment in the grafts in syngeneic rat intraportal islet transplantation models (19–21).

The NF-κB signaling pathway downstream of TNFR, IL-1R, and TLR4 plays an important role in the innate immune response. Macrophages are activated through the NF-κB pathway and release proinflammatory cytokines such as TNF-α, IL-1, IL-6, and IL-12. The NF-κB signaling pathway is regulated by TNFR1, IL-1R, TLR, and other receptors, so the regulation of the NF-κB signaling pathway is the focus of research on immune tolerance after islet transplantation. Dehydroxymethylepoxyquinomicin acts as an inhibitor of NF-κB to inhibit NF-κB activation at the nuclear translocation level. It can inhibit the activation of TNF-α, IL-6, and serum high mobility complex-1 on macrophage-based immune cells through the NF-κB pathway and protect islet grafts from the injury caused by transplantation via the portal vein (22, 72).

**Figure 2:**
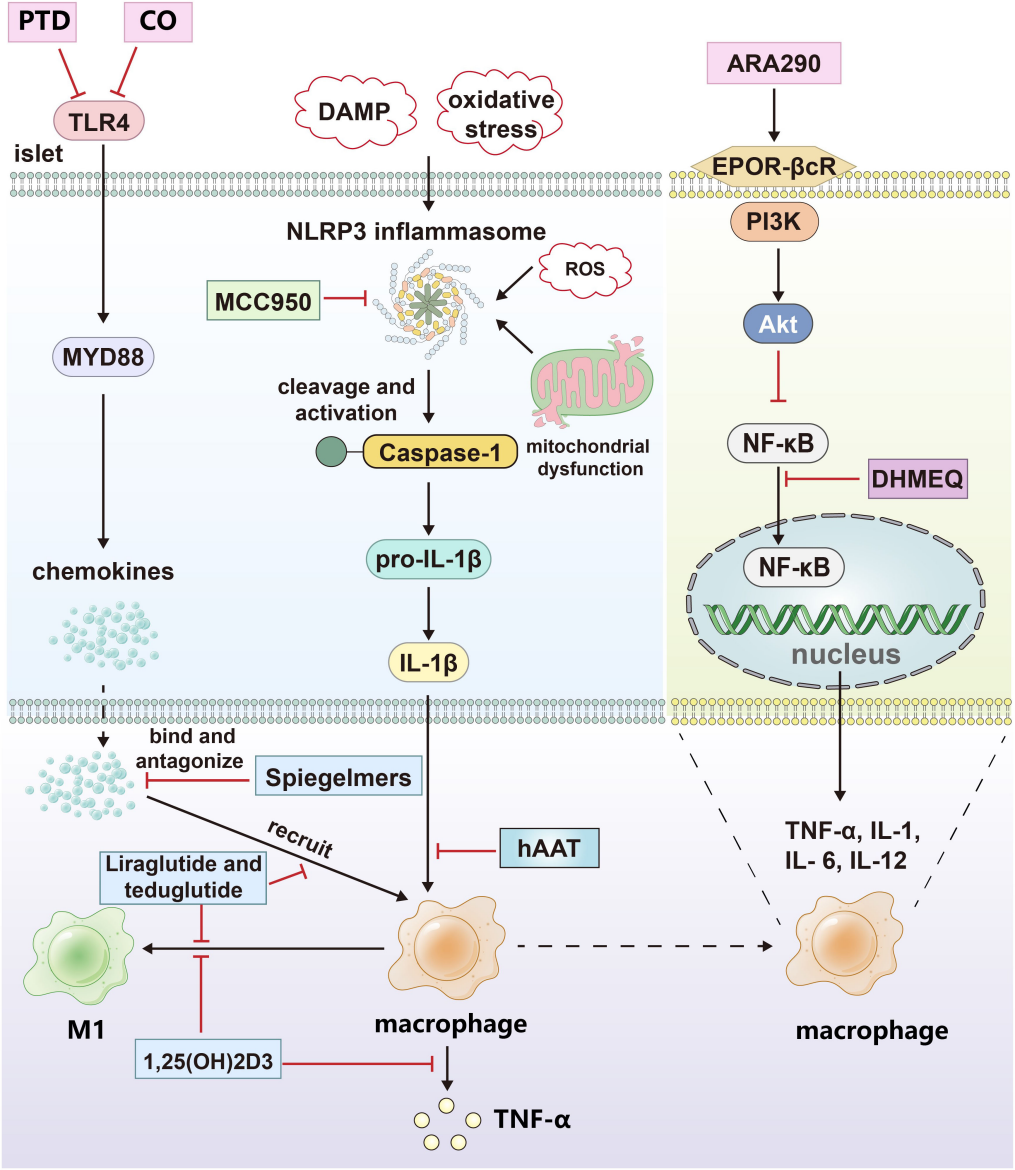
The drug treatment strategies targeting islets and macrophages. CO and protein transduction domain proteins (PTD) blocked the signal pathway of TLR4; Spiegelmers binds to and antagonizes chemokines; MCC950 inhibits the secretion of IL-1β by inhibiting NLRP3 inflammasome. hAAT inhibits the effect of IL-1β by increasing the expression and secretion of IL-1Ra in primary islets and macrophages. Calcitriol inhibited the secretion of proinflammatory factors and M1 polarization of macrophages; Dehydroxymethylepoxyquinomicin (DHMEQ) inhibited the nuclear translocation of NF-κB; ARA290 blocked NF-κB pathway by activating EPOR-βcR/PI3K-Akt signal pathway; Liraglutide and teduglutide inhibit the expression of proinflammatory factors and M1 polarization in macrophages. The black arrows indicate promotion and the red inhibitors indicate inhibition.

ARA290, a pyroglutamate helix B surface peptide composed of 11 amino acids particular to EPOR-βcR without the side effects of erythropoietin (EPO), promotes endothelial cell activation and platelet reactivity. Through the activation of the EPOR-βcR/PI3K-Akt signaling pathway, ARA290 can inhibit the transcription of proinflammatory factors driven by the NF-κB pathway and the activation of macrophages; reduce the transcription levels of IL-6, MCP-1, MIP-1β, TNF-α and IL-1β in the graft; and thus prohibit the damage of proinflammatory factors on islets and the proapoptotic effect, thereby improving blood glucose levels and islet graft function (23). Similarly, the application of liraglutide, an analog of GLP-1, and teduglutide, a glucagon-like peptide-2 receptor agonist, can inhibit the expression of proinflammatory cytokines, macrophage recruitment, and M1 phenotype polarization during and after transplantation and can thus improve graft function. Its ability to polarize macrophages may be mediated by the cAMP-PKA-STAT3 signaling pathway (24–26) (**Figure 2**).

Currently, significant progress has been made in drug trials for macrophages. However, most experiments often use experimental drugs individually, neglecting the potential impact of current clinical immunosuppression schemes on macrophages. After years of development, the immunomodulation protocol improved by the Edmonton protocol, which includes tacrolimus and mycophenolate mofetil combined with dalizumab or baliximab, has been widely applied to islet transplantation in most transplant centers around the world (73). As a calcineurin inhibitor, Tacrolimus can inhibit T cell activation and cytokine production. It has been found that tacrolimus inhibits the activation of the JAK2/STAT3 signal by targeting JAK2, thereby inhibiting the polarization of M2 macrophages and exerting its anti-fibrosis function (74). After human islets were transplanted into NSG mice, tacrolimus activates islet resident macrophages by inhibiting the NFAT pathway and stimulates them to produce IL-1β by increasing amyloid deposition in transplanted islets, thus inhibiting β cell function. The application of Exendin-4 reduces this effect (75). In recent years, the potential effects of mycophenolate mofetil on monocytes have also been discovered, including the inhibition of proinflammatory factor secretion and adhesion molecule expression. Baliximab can upregulate the percentage of CD14+CD163+ monocytes (76). At present, there are few studies on the influence of existing immunomodulation schemes for islet transplantation on macrophages. Ignoring this potential influence may pose risks in clinical trials and lead to inconsistencies between therapeutic effects and animal experiments. Therefore, fully integrating drug research on macrophage immunomodulation into clinical practice is imperative.

### 4.2 Regulation of macrophages in islet transplantation by ECM and interstitial cells

Islet transplantation, unlike liver, kidney, and pancreas transplantation, falls more into the category of cell transplantation. Therefore, in addition to regulating the recipient’s immune system, research on islets has also become a focus of immune regulation.

#### 4.2.1 The role of ECM in immune regulation

The islet mass typically accounts for 2% of the total pancreatic mass, and a boundary composed mainly of the ECM usually exists between the endocrine islet cells and the exocrine acinus. During the islet isolation process, the digestion of enzymes can disrupt the ECM, thus destroying the structure and function of the islet. In addition, the concept of the “capsule” of the islet, which describes the stromal structures surrounding the islets, has recently arisen. There is a population of resident macrophages enriched in the peri-islet capsular area that play a barrier role in preventing the infiltration of other immune cells in the disease state, so maintaining the integrity of the interstitial structure around the islets is vital for maintaining the therapeutic efficacy of islet transplants (77).

Type IV and VI collagen proteins and laminin are essential components of the islet ECM. Matrix metalloproteases are a family of zinc-dependent endopeptidases involved in ECM turnover under several conditions. MMP-2 (gelatinase A) and MMP-9 (gelatinase B) interact with elastin and types I, III, and IV gelatins when activated, facilitating their degradation within connective tissue matrices. In islet transplantation, macrophages and neutrophils can secrete gelatinases, such as MMP-9, which can degrade ECM. The knockdown of MMP-9 and application of the MMP inhibitor captopril can significantly reduce macrophage infiltration into the islets, thus protecting the function of the graft and prolonging its survival time (27). However, another study showed that after mouse islets were transplanted into recipient muscle tissue, VEGF-A, expressed at high levels, could recruit CD11b+/Gr-1+/CXCR4hi neutrophils and induce this neutrophil subset to secrete a large amount of the effector protein MMP-9. However, islet revascularization was impaired in MMP-9-deficient mice. Thus, MMP-9 is essential for vascular reconstruction and functional integration following islet transplantation (78). Although therapeutic strategies targeting MMP-9 can reduce the degradation of ECM and the infiltration of inflammatory cells, mainly macrophages in the early stage, MMP-9 knockdown or inhibitors affect the vascularization of graft islets in the late stage. Therefore, the application of MMP-9 knockout (KO) or inhibition is highly controversial. The protection of ECM integrity and reduction of inflammatory cell infiltration without affecting the process of islet vasculogenesis remain challenging.

In addition to maintaining ECM integrity, the inhibition of the attachment of leukocyte and platelet aggregates to islets after ECM destruction is also a promising research direction. Diannexin is a recombinant homodimer of the endogenous anticoagulant molecule annexin V, which binds externalized phosphatidylserine residues on the surface of early apoptotic cells, thereby suppressing the attachment of leukocytes and platelet aggregates. In the syngeneic mouse renal subepithelial transplantation model, diannexin application reduced the recruitment of macrophages and T cells to the islet periphery, significantly reduced the mRNA expression level of the apoptotic marker Bid, reduced islet cell apoptosis, and improved the early function of islet transplants (28). Although diannexin has already undergone phase II clinical trials in kidney transplantation and achieved outstanding results in the early stage of posttransplantation, further exploration is needed to determine its efficacy after portal vein transplantation due to the graft in the portal vein comes into direct contact with a large amount of blood.

Additionally, this study did not demonstrate any advantage in using diannexin during the late stage of posttransplantation. Is this due to a reduction in macrophage recruitment during the early stage, which may potentially damage the vascularization process of the graft? The validation of this issue necessitates further investigation in subsequent studies.

#### 4.2.2 Cotransplantation of islets and MSCs

Islet transplantation offers an additional advantage in that the islet preparation can be augmented with supplementary cells possessing immunomodulatory properties, thereby regulating the immune response following transplantation. Mesenchymal stem cells (MSCs) are self-renewing multipotent mesenchymal stromal cells that can be isolated from tissues of mesodermal origin and can differentiate into a cell lineage. MSCs have been widely used in the field of autoimmune diseases and transplantation due to their low immunogenicity and ability to affect innate and specific immune cells through the release of various immunomodulatory factors. The low immunogenicity of MSCs is due to the lack of expression of major histocompatibility complex-II molecules on their cell surface and the lack of expression of costimulatory molecules (CD40, CD86, or CD80) important for immune recognition. Therefore, the application of allogeneic MSCs would not cause severe immune reactions in recipients (79). By far, the most prevalent source of MSCs in clinical trials is adult bone marrow, followed by adipose tissue and puerperal discards such as umbilical cord tissue and placental cells (80). In islet transplantation, MSCs can promote the polarization of mononuclear cells/macrophages to the M2 phenotype by secreting exosomes and cytokines including Indoleamine 2,3-dioxygenase (IDO), IL-4 and IL-10 (81), while inhibiting their differentiation to the M1 phenotype, reducing the production of IL-12 and blocking the maturation of dendritic cells; the ability of immature dendritic cells to present antigen will be diminished and ultimately weaken T-cell function, thus improving the survival rate of the transplanted islet (82). At present, the MSC research directions are mainly the selection of subgroups under different types and states and MSC engineering.

Adipose-derived mesenchymal stem cells (ASCs) have advantages such as easy acquisition, more extensive multipotential differentiation ability, a better immunoregulatory effect, and more secretion of angiogenic factors (83, 84). Innate immune responses are the leading cause of transplant failure for patients undergoing total pancreas resection and autologous islet transplantation. ASCs derived from chronic pancreatitis patients show no significant differences in phenotype, differentiation capacity, or secretion of growth factors compared to those derived from healthy donors. The cotransplantation of mouse islets and chronic pancreatitis-ASCs can inhibit the expression of TNF-α and Bcl-2 modifying factor. Chronic pancreatitis-ASCs mediate the expression of the graft anti-apoptotic gene TNF receptor superfamily member 11b through paracrine IGF-1 and reduce the infiltration of macrophages into the graft and β-cell death, ultimately improve islet function (29). This study has some implications for clinical islet autotransplantation because, although MSCs have low immunogenicity, they also express MHC I molecules and MHC II will be expressed under the influence of IFN-γ (85). Therefore, cotransplantation of the recipient’s own ASCs would be a better choice if clinical conditions permit. More encouragingly, the clinical trial of autologous MSCs and islet cotransplantation has been initially carried out, improving transplant patients’ prognosis based on sound safety (30).

Given the potent immunomodulatory effects of hAAT, the cotransplantation of hAAT-engineered mesenchymal stromal cells and islets was shown to inhibit macrophage migration, significantly reduce the infiltration of CD11c+ and F4/80+ cells, and increase the number of CD206+ cells. By transforming macrophages into a protective state favoring islet survival, hAAT-engineered mesenchymal stromal cells significantly improved the survival of cotransplanted islets (86).

However, there are limitations in the application of MSCs at present, and the protective effect of MSCs on the graft is partly achieved through direct contact with the graft and the cytokines it secretes (87). Ensuring close contact between MSCs and islets during transplantation into the recipient’s liver is challenging, and there is also a risk of MSC-induced thrombosis in the liver (88). Cotransplantation of MSCs and islets under the renal capsule can solve this problem. Still, this transplantation method has the disadvantages of difficult transplantation and systemic insulin release, so it is difficult to apply to clinical work. Therefore, a team has proposed a method of carrying immunomodulatory components derived from MSCs on islet microcapsules. The MSCs-derived exosomes have the immunoregulatory ability to multiply immune cells. After being loaded on the islet microcapsule, they can exert their immunoregulatory ability on macrophages by regulating the NF-κB signaling pathway, thus alleviating pericapsular growth and fibrosis and significantly delaying the rejection of xenogeneic islets in mice recipients (31). Although this study did not adopt a portal vein transplantation model, it still provides insights into the cotransplantation of MSCs.

### 4.3 Optimization of islet cells

Islet transplantation is a minimally invasive organ transplantation technology. The technical key and difficulty lie in the isolation and optimization of islet cells and the development of and breakthroughs in transplantation technology.

#### 4.3.1 Optimization of islet isolation and culture techniques

The islet preparation protocol used in clinical practice is becoming increasingly mature; however, research on its optimization is ongoing. Reducing islet damage during preparation can yield high-quality islets and minimize the innate immune response after transplantation.

##### 4.3.1.1 Optimization of islet isolation

Islets are cell masses with a diameter of 100 to 400 μm, and the size of the islet cell mass can affect the outcome after transplantation. In one study, islets with a mean diameter of 250 μm were divided into a small islet group (mean diameter <250 μm) and a large islet group (mean diameter >250 μm). The small islet group showed higher insulin secretion and viability and lower levels of microthrombosis, inflammatory cytokine expression, and inflammatory cell infiltration after transplantation (89). Although further selection of isolated islets will waste clinical resources in the context of the shortage of donor pancreas, this study provides some enlightenment for the strategy of differentiation of islet cells by inducing iPSCs.

Islet purification is the process of separating isolated islet cells from other cells in the pancreatic parenchyma, such as exocrine acinar cells and interstitial cells, to obtain a higher purity islet cell preparation. High-purity islet cell preparation can reduce complications such as increased portal vein pressure during transplantation, the innate immune response after transplantation, and the infiltration of neutrophils and macrophages. In clinical practice, islet purification is usually carried out by continuous gradient density centrifugation using Ficoll solution with different densities and a COBE2991 centrifuge. The purification method based on iodixanol (OptiPrep) has been applied in experimental research and clinical applications. Compared with Ficoll purification, the efficiency of islet purification by this method was not significantly different. The production of cytokines/chemokines such as IFN-γ, TNF-α, IL-1β, IL-6, IL-8, RANTES, MCP-1, and MIP-1β in the supernatants of islet preparations in the OptiPrep group was significantly reduced after 48 hours of culture (32). The reduction in cytokines/chemokines can significantly attenuate the infiltration of immune cells, such as macrophages, after transplantation.

##### 4.3.1.2 Optimization of islet culture

Another procedure before islet transplantation is islet culturing and preparation of islet preparations, which is of particular importance for the transportation of islet preparations to interregional clinical transplant centers. On the other hand, the culture before transplantation creates conditions for the quality control of the preparation and the immune induction of the recipients before transplantation. Most importantly, a period of culture can improve purification quality, reduce the number of apoptotic cells and byproducts, and thus attenuate the innate immune response after transplantation (7).

The optimization of culture methods can further reduce the damage to isolated islets. For example, an islet culture medium with fibrin as a scaffold and perfluorodecalin as an oxygen diffusion-enhancing medium was shown to improve islet function, islet viability, and islet cell hypoxia caused by three-dimensional medium encapsulation (90). However, the islets cultured by this method caused an infiltration of macrophages around the graft in the early stage of transplantation into the portal vein of rats, which may have been caused by the attachment of matrix residues to integrins and the acceleration and enhancement of the IBMIR (91). Therefore, developing an alternative scheme to current *in vitro* islet culturing to increase the survival rate of islets while minimizing the introduction of additional antigens to reduce the infiltration of macrophages around the transplant is a promising research direction.

Adding drugs to the islet culture process to inhibit islet activation and thereby reduce macrophage recruitment after transplantation is also viable.

The complement cascade plays an amplifying role in the IBMIR, with the core step being the cleavage of C3 into C3b by the C3 convertase. This ultimately leads to the assembly of the membrane attack complex, the release of soluble C3a and C5a, and thus the activation and recruitment of inflammatory cells. APT070, also known as mirococept, is a modified fragment of complement receptor 1 (CD35) that can protect cells against complement activation. By preincubation with islets, C-peptide release, iC3b production, and C4d and C5b-9 deposition in islets embedded with thrombi were reduced *in vitro*. In a humanized mouse renal subcapsular islet transplantation model, APT070 reduced the infiltration of human CD45+ cells, macrophages (CD11b+), and neutrophils (CD66b+) into the islets (33).

Human intestinal amyloid precursor protein (hIAPP) is a 37-amino-acid peptide cosecreted with insulin by β cells (92) that can promote the recruitment of macrophages by inducing the secretion of chemokines, such as CCL2 and CXCL1, and induce macrophages to secrete proinflammatory factors such as TNF-α through the IL-1R/MyD88 pathway. hIAPP is cytotoxic and induces β-cell apoptosis through the IL-1β/Fas/caspase-8 apoptotic pathway, which is an important reason for long-term transplant failure (93, 94). In the pretransplantation culturing process, applying the IL-1Ra anakinra can reduce the formation of hIAPP, reduce macrophage infiltration and antagonize hIAPP-induced β-cell apoptosis (34). Although anakinra has been used in the clinical treatment of rheumatoid arthritis and, when combined with etanercept, can improve islet function (3), there have been clinical reports of subcutaneous amyloidosis caused by long-term subcutaneous injection of anakinra (95). Therefore, further observation is still needed to determine its long-term efficacy. Optimized islet culture techniques can reduce the infiltration of inflammatory cells such as macrophages in clinical transplantation, prolong the *in vitro* survival time of separated islets, reduce the preparation frequency of islet cells for research, and provide excellent convenience for experimental research on islet cells.

#### 4.3.2 Related studies on the engineering of islet cells

In addition to optimizing the preparation process of islets to reduce their immunogenicity, another strategy to reduce inflammation and macrophage regulation after transplantation is to engineer islet cells.

##### 4.3.2.1 Gene modification of islets

Gene modification technology has been widely used in the field of organ transplantation, especially in the field of xenotransplantation. Specific gene KO animal-derived islets can achieve better transplantation results in islet transplantation.

Activation transcription factor 3 (ATF3) is a stress-induced apoptotic gene whose expression is upregulated by various signals during islet isolation and transplantation, such as cytokines, nutritional deficiency, serum stimulation, and calcium signals. After transplantation, the infiltration of macrophages into the grafts was found to be significantly reduced in islets taken from ATF3 KO mice. The expression of caspase-3 and apoptotic factors (Noxa, bNIP3) in transplants was significantly reduced, and the grafts had better glucose homeostasis (35).

KO corresponds to a specific immunosuppressive gene overexpression strategy. Developmental endothelial locus-1 (Del-1) is an endothelium-derived anti-inflammatory glycoprotein that regulates β2 integrin-dependent leukocyte adhesion. In a syngeneic portal vein graft model using mice with endothelial cell-specific overexpression of Del-1 as recipients, the overexpression of Del-1 inhibited platelet-monocyte aggregate formation by the binding of the leukocyte β2-integrin Mac-1 to cognate counterreceptors on platelets, predominantly glycoprotein Ib. The infiltration of Ly6G-CD11b+ cells (monocytes) in the liver was reduced, thereby reducing the intensity of the IBMIR and protecting the islets from damage (36). Similarly, the knock-in of immunosuppressive genes is a promising way to modify islets.

Heme oxygenase-1 (HO-1) has been identified as a ubiquitous stress protein with antioxidant, anti-apoptotic and anti-inflammatory effects and improved outcomes of islet allografts and xenografts. Soluble TNF-α type I receptor (sTNF-αR) can inhibit TNF-α-induced cell activation. An adenovirus vector was used to overexpress the fusion protein sTNF-αR-Fc/HO-1 of human HO-1, sTNF-αR, and human IgG1Fc in porcine islets. The modified porcine islets transplanted into the subrenal capsule of humanized mice significantly inhibited the infiltration of macrophages and T cells into the grafts. It also reduced the expression of RANTES, TNF-α, and IL-6 and inhibited the apoptosis of grafts (37).

Due to the shortage of donor pancreases, inducing iPSCs to differentiate into islets has become a prominent research topic. However, stem cell-derived islets’ potential cytotoxicity or oncogenicity is the main problem. Additionally, for patients with autoimmune diabetes, islets differentiated from autologous stem cells are also at risk of being attacked by autoimmunity. Gene modification technology serves as an effective means to address these issues (96). In recent research, primary human islet cells can escape the killing effect of macrophages, NK cells and subsequent adaptive immune response in humanized mouse model through KO the genes encoding class I and II MHC and over express CD47. Furthermore, through gene modification of T1DM patients’ iPSC-derived islets, autoimmune escape was realized in autologous, diabetic humanized mice. By blocking CD47, the islet in the recipient can be eliminated, which ensures the safety of clinical application in the future (38). This study suggests that reserving the “switch” to remove the modified cells can greatly improve the safety of gene editing technology.

##### 4.3.2.2 The application of material chemistry in islet transplantation

With the continuous development of materials science and chemistry technology, breakthrough progress has been made in its application in the medical field, including but not limited to drug delivery and tumor targeting. Choosing a suitable material to encapsulate islet cells and isolate islets from the portal microenvironment can block the innate immune response and improve the patient’s long-term dependence on immunosuppressants. However, the difficulty of islet microencapsulation is avoiding contact between islets and the blood while ensuring the exchange of oxygen and nutrients and allowing the hormones released by islets to enter the circulation. Another problem of microencapsulation is that although the capsule of the islet prevents the infiltration of inflammatory cells such as macrophages into the graft, the capsule itself as a foreign body will also cause the infiltration of inflammatory cells, and long-term macrophage infiltration will cause fibrosis around the capsule. The capsule structure itself is also an obstacle to vascularizing the islet. The hypovascularization of the graft will interfere with the local clearance of hIAPP. The presence of hIAPP in the encapsulated transplant will lead to graft failure (97).

In previous studies, alginate has always been a focal point in the research on microcapsule materials due to its excellent biocompatibility and ease of preparation. However, the variability in alginate production results in inconsistent endotoxin content and purity, which can affect its biocompatibility as microcapsules (98). Moreover, microcapsules formed by alginate-based hydrogels will allow unnecessary cells to enter and exit due to their intrinsic softness and open network structure. In contrast, microcapsules made of traditional polymers such as polytetrafluoroethylene or polycaprolactone can effectively prevent cell escape but may lead to pericapsular fibrosis (99). Unlike the microcapsules made from the materials mentioned above, thermoplastic polyurethane-based nanofiber capsules can minimize foreign body reactions and block unwanted macrophage activation (39). Another advantage of nano-materials is that the shape of microcapsules, the size of pores and the density of pores can be designed. Generally, globular proteins have a diameter ranging from 2 nm to 10 nm, while organic metabolites have a diameter between 0.5 nm and 1 nm. Immune cells like macrophages and leukocytes are approximately 6-10 microns in diameter; therefore, microcapsules with a pore size of 20 nm can maintain cell function and reduce essential immune components (100). However, the potential cytotoxicity of nano-material microcapsules is a problem that cannot be ignored, and further large-scale non-human primate experiments and clinical trials are necessary to verify their safety (101).

Another strategy for islet encapsulation is drug loading of the capsule. Dexamethasone (Dex) is an immunosuppressive glucocorticoid that effectively inhibits inflammatory pathways and can polarize human blood-derived monocytes to the M2 phenotype while preserving their migratory function. However, in contrast to other organ transplants, higher concentrations of Dex in islet transplants severely impair cell mobility and lead to impaired engraftment and angiogenesis. It also impairs the glucose responsiveness of β cells. A polydimethylsiloxane scaffold equipped with Dex can locally deliver immunoregulatory Dex in a controlled manner and polarize macrophages into the M2 phenotype without affecting islet function, thus creating a protective microenvironment for transplanted islets (102). Similarly, the transplantation of dexamethasone 21-phosphate (Dexa)-containing chitosan-coated alginate microencapsulated porcine islets into the enterocoelia of diabetic mice can reduce macrophage-dominant inflammatory cell infiltration and pericapsular fibrosis (40). These results suggest that microencapsulated islet transplantation strategies have great potential in islet xenotransplantation.

In recent years, bilirubin, as the end product of heme metabolism, has been found to inhibit the infiltration of KCs into transplanted islets in the liver (103). On the other hand, bilirubin can activate the Nrf2 pathway to polarize macrophages to the M2 phenotype and inhibit the NF-κB pathway to inhibit M1 polarization and enhance the function of M2 macrophages, thus playing an antioxidant and anti-inflammatory role. ϵ-Polylysine-bilirubin conjugate-encapsulated islets can effectively promote the polarization of macrophages to the M2 phenotype, optimize the immune microenvironment for islet survival and function, and maintain the normal blood glucose level of recipients for more than 35 days (41).

**Figure 3:**
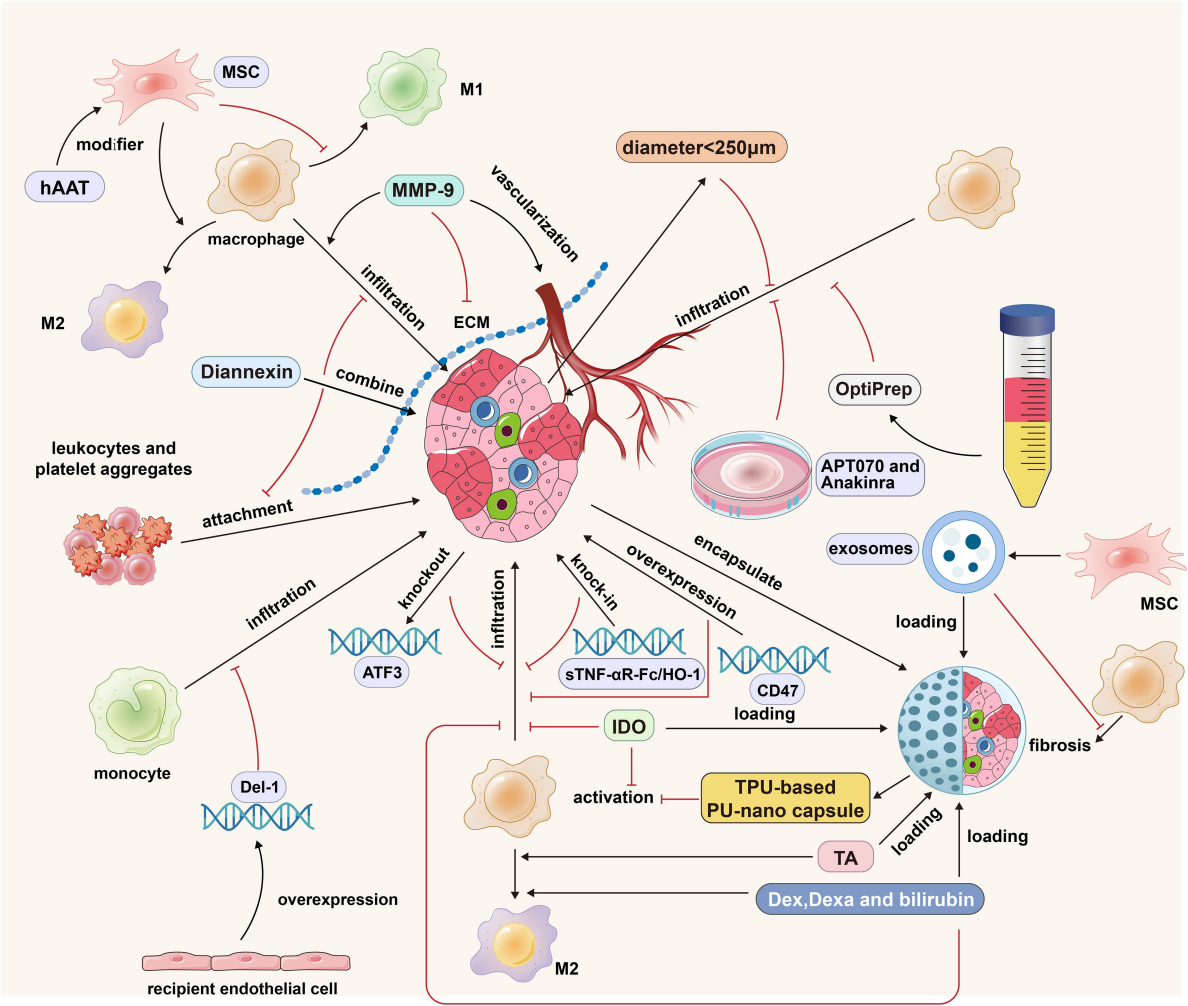
The strategies of islet cells engineering, islet isolation optimization, islet culture optimization, ECM regulation and MSCs cotransplantation in islet transplantation. MMP-9 promotes the infiltration of macrophages into the graft by degrading ECM, but it also has the function of promoting the vascularization of the graft; Diannexin inhibits the adhesion of leukocytes and platelet aggregates by binding to the externalized phosphatidylserine residues on the islet surface; MSCs promote the polarization of M1 macrophages into M2 macrophages; The modification of hAAT strengthened the anti-inflammatory effect of MSCs; The islet diameter less than 250 microns and the purification method based on OptiPrep can reduce the expression of graft cytokines/chemokines and reduce the infiltration of immune cells; APT070 can inhibit the activation of complement system and the infiltration of immune cells; Anakinra can reduce the formation of hIAPP, thus reducing macrophage infiltration; Donor ATF3 KO, sTNF-αR-Fc/HO-1 overexpression and receptor Del-1 overexpression can reduce the infiltration of mononuclear macrophages; Thermoplastic polyurethane-based nanofiber capsules can inhibit the activation of macrophages; Microcapsule-loaded Dex/Dexa/bilirubin can promote the M2 polarization of macrophages and reduce the infiltration of macrophages into the graft. The black arrows indicate promotion and the red inhibitors indicate inhibition.

IDO is a cytosolic, heme-containing enzyme that catalyzes the first and rate-limiting step in metabolizing the essential amino acid L-tryptophan to N-formyl kynurenine. Its high expression is one of the causes of tumor immune escape, and its immunoprotective effect on T-cell-mediated allogeneic rejection has been widely studied in organ transplantation. An islet-fibroblast composite graft composed of Indoleamine 2,3-dioxygenase-expressing fibroblast-populated collagen gel matrices and islet cells can induce tryptophan deficiency, reduce the viability of macrophages, inhibit their proinflammatory activity, and reduce the infiltration of macrophages in the graft (42). Tannic acid (TA) is a natural polyphenol with antioxidant activity that can scavenge free radicals, inhibit free radical-induced oxidation, and elicit immunomodulation. A novel cytoprotective nanothin multilayer coating for islet encapsulation consisting of TA and poly(N-vinylpyrrolidone) can reduce M1 macrophage polarization and the chemokine synthesis involved in leukocyte recruitment and increase the expression of alternatively activated M2 macrophage-associated mRNAs, such as Arg1, Retnla and Ccl17, in the graft. The frequency and cell number of Arg-1+ and CD206+ macrophages are significantly increased, alleviating islet transplantation rejection (43–45). In summary, drug encapsulation is not only used as a supplementary method for islet microencapsulation but also has the advantages of reducing the immunogenicity of the capsule material itself and delivering drugs to the target area in a localized and controlled manner, which can regulate the polarization of macrophages while minimizing the systemic adverse reactions and side effects of drugs. However, loading drugs onto microcapsules limits the total amount of drugs that can be loaded. How do we deliver drugs targeted when the drugs loaded are exhausted? Furthermore, verifying whether grafts that lose the immunomodulatory effect of drugs can maintain long-term function is necessary. Additionally, most related studies do not utilize portal vein transplantation, and different transplantation sites may result in variations in graft function (**Figure 3**).

## 5 Discussion and outlook

Islet transplantation, an emerging organ transplantation technology, solves the problem of insufficient insulin secretion and dramatically improves the quality of life of diabetic patients. Inhibiting the proinflammatory effect of macrophages in the IBMIR directly or indirectly through different therapeutic means can significantly improve the survival rate of early grafts. However, macrophages have a “double-edged sword” role in the inflammatory response. M2 macrophages have the potential to promote the proliferation of β cells and, at the same time, play an active role in tissue repair and blood supply reconstruction of the graft (104). Simply inhibiting the infiltration of macrophages into grafts is not conducive to graft repair and the vascularization process. Regulating the M1/M2 phenotype of macrophages is the most prevalent treatment strategy currently employed, which can mitigate the detrimental effects of inflammatory responses on grafts without compromising the functional capacity of M2 macrophages. However, on the one hand, the potential inhibitory effects of this approach on subsets of cells that are beneficial to graft function remain unclear. On the other hand, many studies have confirmed the role of M2 macrophages in renal and pulmonary fibrosis. M2 macrophages promote the proliferation and activation of fibroblasts by secreting cytokines and differentiate into αSMA+ myofibroblasts through a process called macrophage-to-myofibrolast transition mediated by TGFβ1–Smad3 signaling. Therefore, it is uncertain whether regulating macrophage polarization will lead to fibrosis of transplanted islets (105–107). In the latest research, mice’s kidney macrophages were divided into monocyte-derived macrophages and kidney resident macrophages by single‐cell RNA sequencing technology after renal ischemia-reperfusion. More importantly, S100a9hiLy6chi monocyte subsets were successfully defined by analyzing tissues and performing RNA velocity analysis during disease progression after renal ischemia-reperfusion, which played a role in initiating and amplifying inflammatory injury throughout the acute phase of acute kidney injury, and were verified in tissue samples from clinical patients. In the mouse model, targeting this subgroup to block S100a8/a9 signaling can effectively prevent acute kidney injury caused by ischemia-reperfusion (108). Further exploration of the heterogeneity of macrophages in inflammation and the targeting of different functional subsets to obtain the most ideal immune tolerance or modulation strategies are promising research directions.

Intraportal islet transplantation is the most common route of islet transplantation in clinical practice. However, in animal experiments, there are other sites that could be used, such as the renal capsule, peritoneal cavity and anterior chamber. These sites may mitigate the IBMIR by reducing islet contact with blood. For example, for patients with T1DM and End-Stage Renal Disease, a new transplantation strategy is to transplant prevascularized islets under the renal capsule of the donor’s kidney. In the non-human primate model, this strategy will not affect the renal function of the donor’s kidney, and it results in better islet function compared to Intraportal transplantation and renal capsule transplantation without prevascularization. Maintaining proper islet function benefits the long-term survival of the donor kidney (109). Compared with the cotransplantation of islets and MSCs, another popular area of research in recent years is the transplantation of islets into MSC-rich tissues. Brown adipose tissue (BAT) is densely vascularized and innervated and is rich in MSCs, M2 macrophages, and immunosuppressive regulatory T cells. BAT is a potential efficacious site for islet transplantation (110, 111). Bone marrow is associated with advantages similar to those of BAT (112). Spleen, an organ rich in MSC, has unique advantages as a transplantation site. On the one hand, insulin produced by transplanted islets can flow into the portal vein through the splenic vein, which is closer to physiological insulin release profiles. On the other hand, spleen has the potential to promote islet regeneration. Although some clinical transplantation centers have performed intrasplenic islet transplantation, there is a risk of arteriovenous thrombosis and subcapsular hematoma (113). A more ideal transplantation site and microenvironment are conducive to the survival and function of the graft. Although these sites have different advantages, none can meet the characteristics of minimal trauma, rich blood supply, and an immune-tolerant environment; at the same time, their safety and effectiveness still need to be verified. Additionally, they are associated with problems such as poor oxygenation and inconsistent outcomes in rodent and large animal islet transplantation models (114). Therefore, exploring new transplantation sites still requires a significant amount of time.

The strategy of combining medical and materials chemistry/engineering techniques can obtain outstanding research results in the field. Islet microencapsulation technology has been a popular research direction in recent years, and the ability of systems created with this technology to carry drugs and release drugs locally highlights its superiority, especially in the field of xenogeneic islet transplantation. Such systems can significantly reduce macrophage-mediated islet destruction and protect graft function. However, pericapsular fibrotic overgrowth is the main problem in this field, and proinflammatory and anti-inflammatory macrophages may mediate this adverse reaction. Only by clarifying the action mechanism of macrophages can we further promote the application of islet microencapsulation technology in clinical work (115). Another advantage of materials chemistry as a delivery system lies in its ability to enhance drug delivery and tissue absorption, while its specific absorption by target tissues solves the side effects of some drugs when administered systemically (116). The strategy of utilizing nano-materials to target liver tissues and regulate the maturation, activation, and polarization of macrophages within the liver has lately garnered significant attention in academic circles.

With the progression of single-cell sequencing and spatial transcriptomics technologies, the identification of previously unknown cell subsets with distinct functions has become feasible. In the future, through a deeper understanding of the role of macrophages in the inflammatory response and the continuous optimization of corresponding immunomodulatory techniques, a more suitable microenvironment for graft survival and function can be constructed, and the prognosis of patients with islet transplants can be gradually improved.

## Author contributions

KD: Writing – original draft. JL: Writing – original draft. JZ: Writing – review & editing. TC: Writing – original draft. HL: Writing – original draft. FL: Writing – original draft. ZL: Writing – review & editing. BG: Writing – original draft. SW: Writing – review & editing. FW: Writing – review & editing.
